# A Case Report Emphasizing the Advantageous Effect of Mulligan Mobilization Technique in the Management of Rotator Cuff Syndrome to Enhance Functional Recovery in a 65-Year-Old Farmer

**DOI:** 10.7759/cureus.63623

**Published:** 2024-07-01

**Authors:** Samiksha V Sonone, Deepali S Patil

**Affiliations:** 1 Musculoskeletal Physiotherapy, Ravi Nair Physiotherapy College, Datta Meghe Institute of Higher Education & Research, Wardha, IND

**Keywords:** mobilization, musculoskeletal physiotherapy, rotator cuff syndrome, rotator cuff tendinopathy, supraspinatus tendinopathy

## Abstract

Adults with shoulder pain often have partial rotator cuff injuries (RCIs) as the underlying cause. RC partial tears are one of the common conditions that can have a major influence on a wide range of people, including sportspeople, workers, and sedentary adults with rotator cuff syndrome (RCS). Any injury, disease, or deteriorating condition that affects the shoulder's RC muscles and tendons is recognized as RCS. Subacromial bursitis, RC tendonitis, subacromial impingement syndrome (SIS), rotator cuff tears (RCTs), etc., are a few disorders linked to RCS. For partial RCT, nonoperative treatment options include physical therapy, anti-inflammatories, analgesics, medication, rest or activity adjustments, and corticosteroid injections. We present the case of a 65-year-old male farmer by occupation, suffering from an RCI on the right side. Following a history of trauma to the right shoulder from a collision with a bull on his farm, the patient complained of pain and limitations in his right shoulder joint. The goal of the rehabilitation program was to maximize the patient's recovery through pain management, range-of-motion (ROM) restoration, muscle strength building, and functional activities. To improve muscular strength and preserve the ROM, strengthening exercises and isometrics were all incorporated into the rehabilitation regimen at the same time. The patient's progress was monitored at scheduled times during rehabilitation using the disabilities of arm, shoulder, and hand (DASH) score, visual analog scale (VAS), goniometer for normal ROM, and the upper extremity functional scale (UEFS). In this case study, the care and recovery of a patient with RC tendinopathy who received physical therapy are examined.

## Introduction

Billions of individuals globally suffer from musculoskeletal injuries, which significantly lower their quality of life. The third most common musculoskeletal ailment is shoulder discomfort, which has a lifespan incidence of roughly 70% and a peak incidence in people aged 40-59 [1]. The shoulder is the most mobile and flexible synovial joint, allowing a huge range of motion (ROM). As a result, shoulder discomfort has a significant socioeconomic impact. The rotator cuff (RC) tendon, lateral and medial elbow epicondyles, gluteal tendons, patellar tendon, and Achilles tendon are the most often affected tendinopathies due to overuse [2]. With over 70% of complaints including shoulder pain, rotator cuff injury (RCI) is the most common reason for this condition. Its symptoms include discomfort with shoulder mobility, particularly when elevating the arm overhead, night-time aching, and also discomfort while sleeping on the side of the lesion, cracking noises when moving the arm, restricted shoulder ROM, and weakening in the RC muscles [3]. Anyone who carries out overhead or repetitive lifting tests runs the additional risk of RCIs. Sportspeople are principally prone to overusing tears, especially when there is evidence of recurrent microtrauma, as seen in baseball pitchers and tennis players [4].

RC biomechanics introduces the term "force couple." As two opposing forces work together to rotate an object, force couples are expressed [5]. The RC muscles stabilize the shoulder joint, granting it both movement and stability. A tear in these muscles results in shoulder impingement, decreased ROM, and joint pain. The valuation process includes a comprehensive medical history and a conversation with the patient. Along with functional and disability questionnaires, screening and continuing observation are also part of it. A valuation of the patient's damages is also part of it, and it may include neural testing, pain behavior, ROM, strength, posterior capsule extensibility, and other conditions on top of that [6]. Acute, traumatic rotator cuff tears (RCTs) usually happen to younger individuals who have experienced a glenohumeral dislocation, falling on an outstretched hand, or clutching an object to catch themselves [7].

There are two approaches to planning the course of treatment for RCIs. For athletes or those with high activity levels, surgery is recommended, particularly for younger patients and those with recent injuries. In a chronic condition, conservative treatment is also recommended. The fundamentals of conservative treatment typically include stages of rehabilitation, physiotherapy, medical care, and protection. It is advised to take it easy and refrain from overhead activities during the protective phase. Steroid injections and nonsteroidal anti-inflammatory medications into the subacromial region help treat the condition medically by reducing discomfort and enabling movement. Applications of platelet-rich plasma (PRP) have been shown in recent years to have a notable and quick healing impact in situations involving RCs, particularly in the early stages. The PRP used in PRP injections has a variety of proteins that promote cell development. Studies comparing PRP application to corticosteroid injection demonstrated that while PRP treatment was more successful in the short term, the two techniques were not longer-lasting. Without a doubt, managing degenerative RCTs involves a nonsurgical approach combined with a suitable physiotherapy program. This is particularly true for individuals who have major surgical risk factors, who choose not to undergo surgery, or who have minor, partial, or irreversible injuries in their bodies [8].

Training the patient about the movement and conditions elements that exacerbate symptoms, physical modalities to lessen or cure symptoms, joint mobilization techniques, stretching and strengthening exercises for muscle weakness or shortness resulting from pain, functionality, or work-related activities are all included in physiotherapy and rehabilitation. Normal ROM, pain reduction, and the restoration of a normal scapula-thoracic rhythm are the objectives. Owing to the increased risk of contracture formation, the most efficient and targeted outcomes come from starting a customized workout program as soon as possible. A mobilization technique called Mulligan's mobilization with movement (MWM) is applied in dealing with musculoskeletal conditions. It requires the patient to actively move the joint at the same time as the therapist glides gently and manually in a prolonged manner. MWM approaches have demonstrated efficacy in mitigating discomfort and enhancing joint ROM in the spine and peripheral joints [9]. 

## Case presentation

Patient information

**Figure 1:**
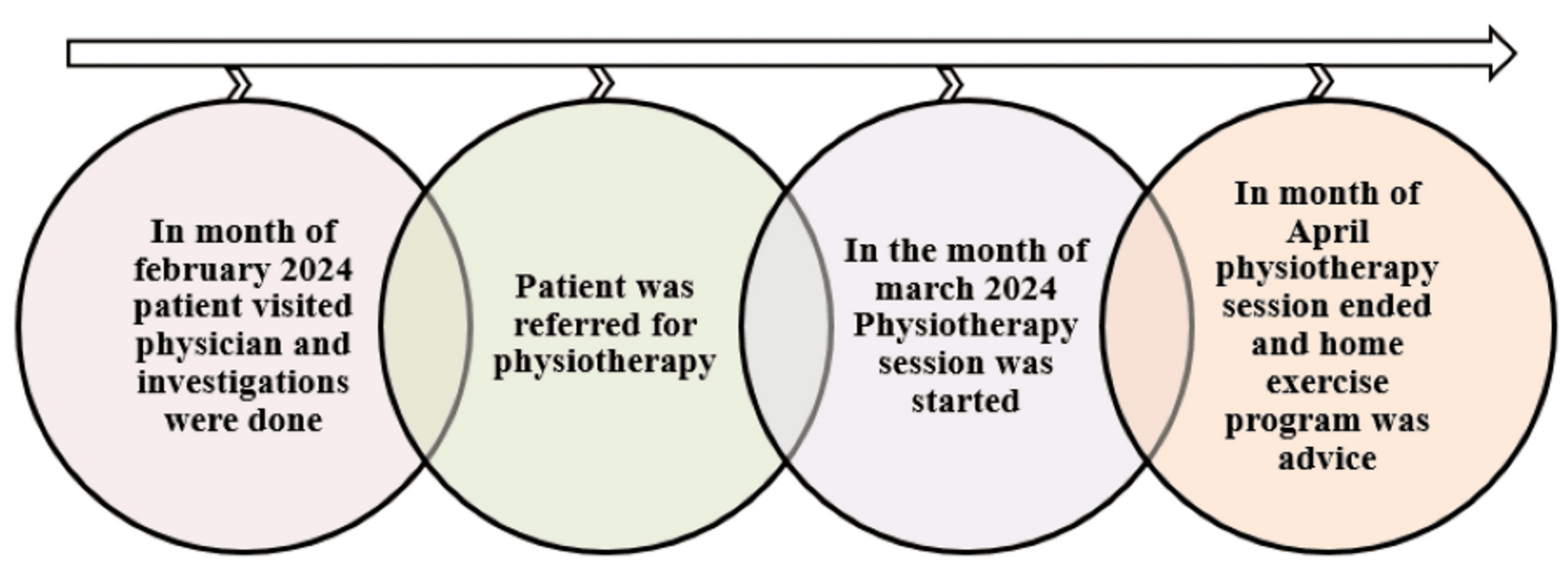
Timeline of events

**Figure 2:**
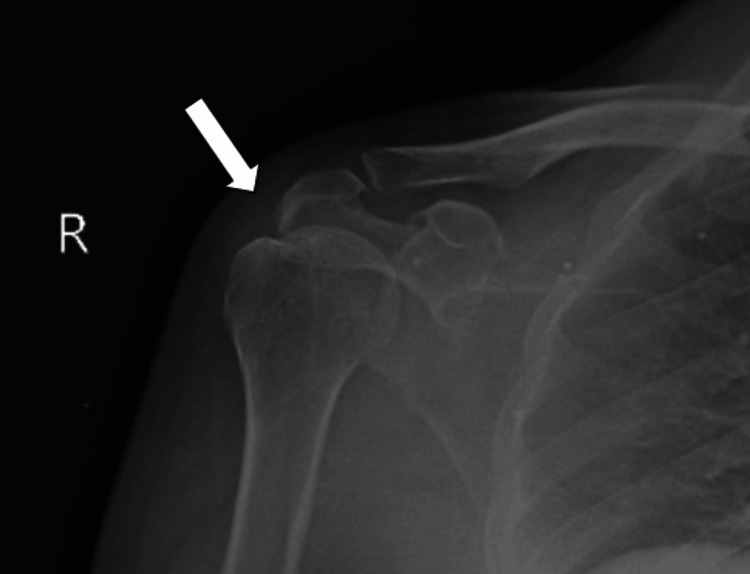
X-ray of the right shoulder joint signifies supraspinatus partial tear The arrow represents supraspinatus muscle partial tear

**Figure 3:**
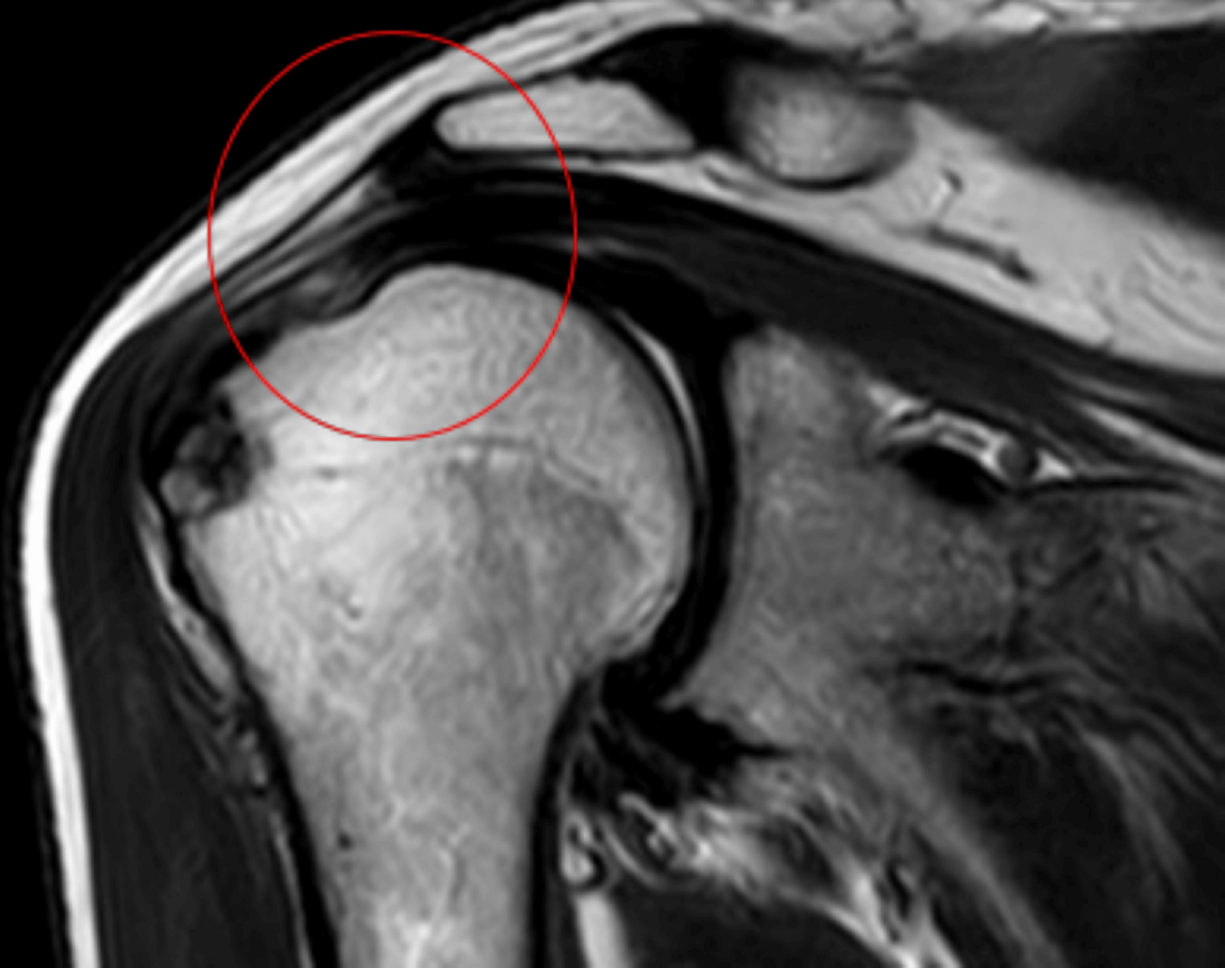
MRI of the right shoulder joint signifies partial tear of the supraspinatus muscle MRI: Magnetic resonance imaging The circle represents a partial tear of the supraspinatus muscle

On history taking, a 65-year-old farmer by occupation narrated that the mechanism of injury was a fall while doing his farming activities. The pain is sudden in onset, dull in nature, and severe in intensity. Pain aggravates with overhead movements and is relieved with rest. As his symptoms were getting worse day by day, he decided to visit a nearby hospital. There, the physician suggested that he should undergo investigation which included X-ray and magnetic resonance imaging (MRI) (X-ray is shown in Figure 2, and MRI, in Figure 3). He was referred for physiotherapy and was prescribed medication. The timeline of events is mentioned in Figure 1. 

Clinical findings

The patient was cooperative and well oriented. Consent was taken from the patient. In clinical diagnosis, the empty can test and belly press test were positive. The patient had limited upper limb mobility and difficulty in overhead activities. ROM of the right shoulder was reduced as compared to the left side mentioned in Table 1 (values of shoulder ROM of the 1st and last physiotherapy session), whereas manual muscle testing of the right side was reduced as compared to the left side mentioned in Table 2 (values of shoulder manual muscle testing on the 1st day and last day of physiotherapy session). Tenderness was present which was grade 3 (patient winces and withdraws the affected parts). On the VAS, discomfort rating was 5.1/10 at rest and on movement 8.7/10, which is mentioned in Figure 5 (outcome measure of pre- and post-physiotherapy intervention). 

**Table 1:** Values of the shoulder range of motion of the 1st and last physiotherapy session

Joints	Movement	1st physiotherapy session	Last (6th week) of physiotherapy session
Shoulder	Flexion	0-75°	0-155°
	Extension	0-20°	0-50°
	Abduction	0-70°	0-145°
	Internal rotation	0-35°	0-55°
	External rotation	0-40°	0-60°

**Table 2:** Values of the shoulder manual muscle testing on the 1st day and last day of physiotherapy session 3-: Movement against gravity greater than one-half range of motion (ROM); 4: movement against gravity with minimal resistance; 5: movement against gravity with maximal resistance

Joints	Muscles	On the 1st day of physiotherapy session	On the last day (6th week) of the physiotherapy session
Shoulder	Flexors	3-/5	4/5
	Extensors	3-/5	4/5
	Abductors	3-/5	4/5
	Adductors	3-/5	4/5
	Internal rotators	3-/5	4/5
	External rotators	3-/5	4/5

Therapeutic intervention

**Figure 4:**
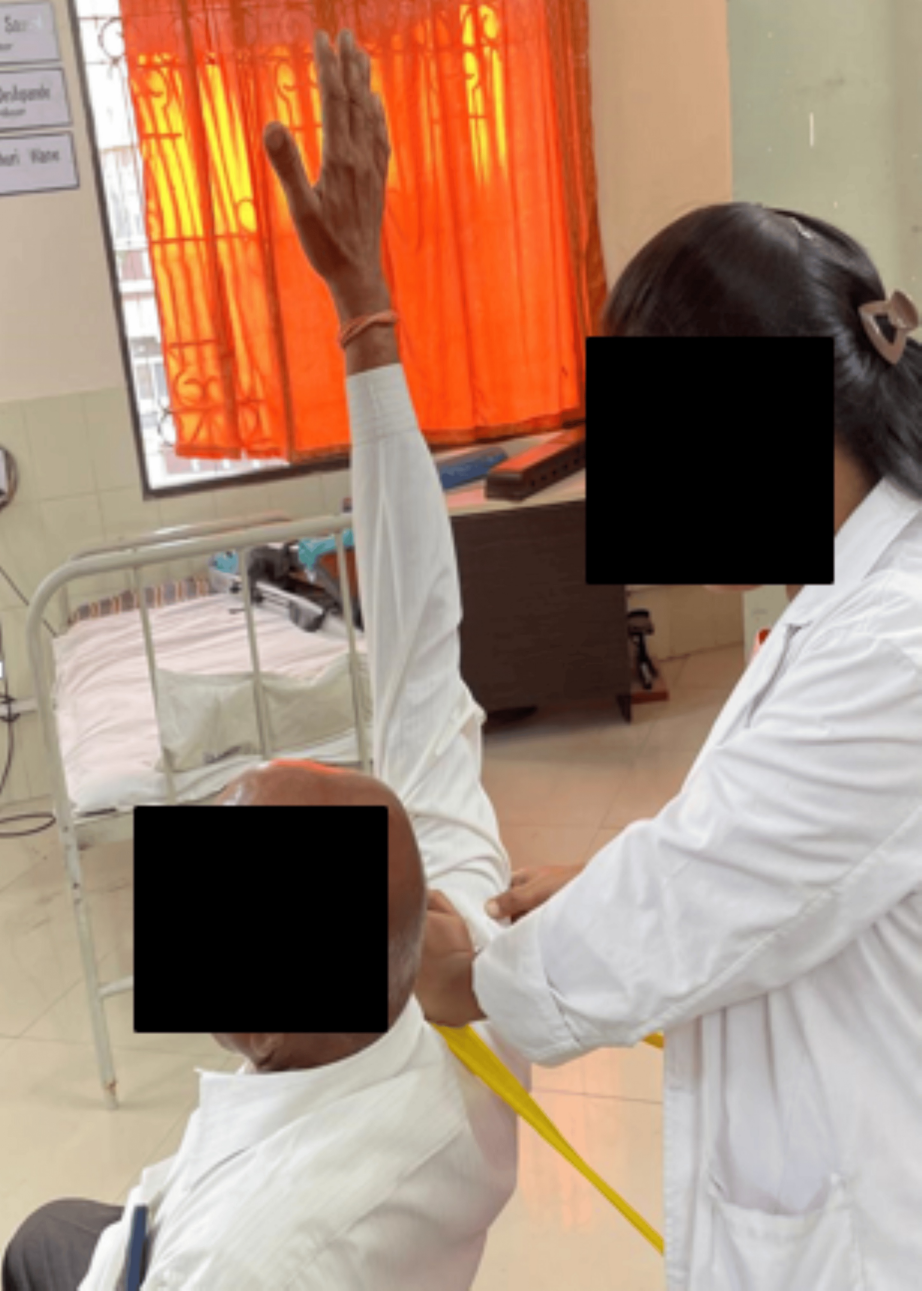
Shoulder abduction mobilization with movement

**Figure 5:**
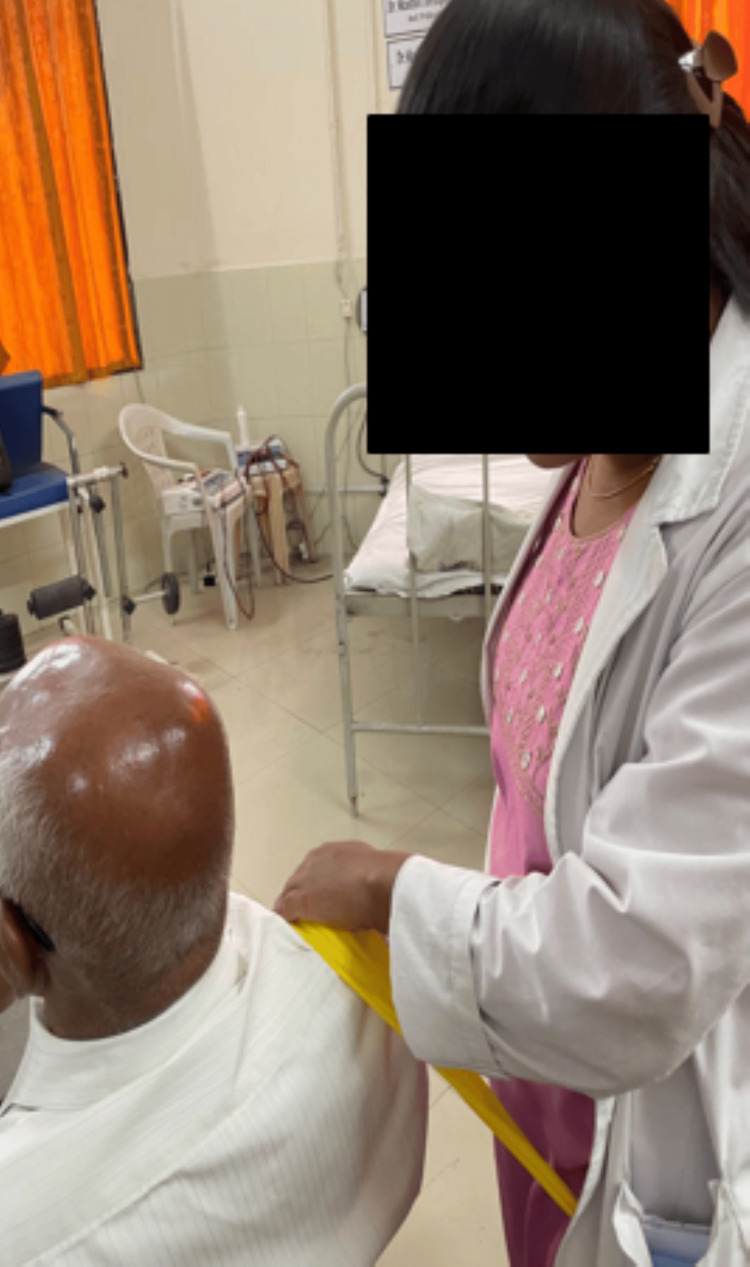
Hand position supporting mobilization belt

A rehabilitation training regime seeks to relieve discomfort, improve mobility, promote long-term recovery, and improve quality of life. Through focused mobilization and strengthening exercises, the major aims include decreasing pain and increasing the range of the RC muscles while treating the underlying causes of RC tendinopathy. Detailed therapeutic intervention according to the patient's problem is mentioned in Table 3, along with their images. Shoulder abduction mobilization with movement is shown in Figure 4, and hand position supporting mobilization belt is shown in Figure 5.

**Table 3:** Detailed therapeutic intervention according to the patient's problem. PROM: Passive range of motion; AAROM: active assisted range of motion; QoL: quality of life; ROM: range of motion; TENS: transcutaneous electrical nerve stimulator; reps: repetition

GOAL	Treatment regimen	Dosage
Patient education	Patients and their relatives received education regarding the injury, the importance of physical therapy, and how it can enhance range of motion, minimize discomfort, and improve quality of life	-
To reduce pain and to promote healing	TENS to reduce pain. Ultrasound to promote healing	Ultrasound of 1.5 MHz in six minutes and TENS of 159 100 Hz in 20 minutes
To improve shoulder ROM	AAROM exercises, such as forward flexion, internal and external rotation, and pendulum exercises, have been carried out within pain limitations	15 reps, three sets/day
To minimize stiffness and increase ROM	The glide included the patient undergoing humeral abduction and the therapist applying overpressure at his end range of motion while a posterior, lateral, inferiorly directed force was applied to the humeral head through the belt	The belt-assisted shoulder abduction MWM consisted of three sets of 10 repetitions (Figure 3)
To improve the strength of shoulder girdle musculature and scapula stabilizers	Shoulder stabilizing muscle protraction-retraction exercises for the scapula in the supine and prone positions and isometric exercises that don't overstress the affected area, respectively	15 reps, five-second hold, three sets/day
To promote anterior and posterior capsule flexibility	Door frame stretching was administered for the anterior capsule, and crossbody and towel stretches were administered for the posterior capsule	30-second stretches, five sets/day

Outcome measure

**Figure 6:**
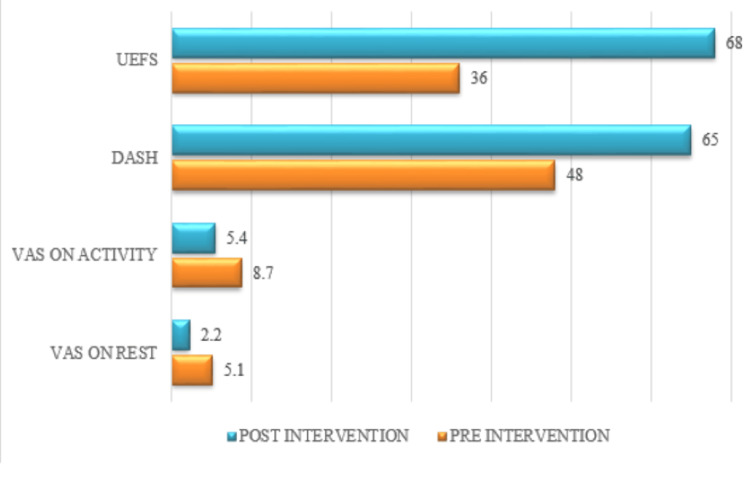
Outcome measures of pre- and post-physiotherapy interventions UEFS: Upper extremity functional scale; DASH: disabilities of the arm, shoulder, and hand; VAS: visual analogue scale The orange color indicates pre-intervention values, whereas the blue color indicates post-intervention values

During physiotherapy treatment, on the 1st day, pre-physiotherapy values were noted, and on the 6th week, post-physiotherapy values were measured. By using the goniometer, the ROM was evaluated (Table 1). The manual muscle test was used to assess muscular strength (Table 2). To evaluate disability of the hand, DASH was used, and to evaluate the functional ability of the hand, UEFS was used. These outcomes are presented in graphical format. Outcome measures of pre- and post-physiotherapy interventions are shown in Figure 6.

## Discussion

Tendinitis is usually treated with rest, anti-inflammatory drugs, and physical therapy. According to research by Edwards et al., RCIs could be successfully managed nonoperatively with physiotherapy. It was critical to recognize that the likelihood of an exercise rehabilitation program being successful is dependent on the patient's responsiveness and recurrence of symptoms. The study's rehabilitation protocol comprised shoulder joint range of motion, door frame and towel stretching to strengthen muscles, scapular protraction-retraction exercises to strengthen the scapular stabilizers, and pendulum exercises [10].

Mulligan mobilization was advantageous in research by Menek et al. that demonstrated the effectiveness of physiotherapy for the nonoperative management of RCIs. In comparison to conventional therapy methods, the study demonstrated that Mulligan mobilization was more helpful for pain, normal joint motion, DASH rating, and some SF-36 characteristics [11].

After RC damage, pain, swelling, and inflammation can be minimized, and normal range of motion can be restored with the use of treatment methods like ice, electrical stimulation, and laser. Studies demonstrating a 32% decrease in external rotation force production and a 23% drop in electromyographic activity in a painful shoulder highlight the importance of prompt pain treatment [12].

According to Zhang's study, the best way to address shoulder joint dysfunction after RCI repair is by combining regular rehabilitation therapies with scapula training activities. Nonetheless, there are clear benefits to increasing scapula training exercises in terms of pain outcomes, activity scope, and overall functional assessment scores [13]. According to a study by Ellenbecker and his colleagues, clinical rehabilitation is the combination of vital physical procedures with evidence-based rehabilitation principles to restore an appropriate range of motion, RC strength, and scapular stability [14]. 

According to Satpute's study, numerous studies have shown that MWM, a type of manual therapy, is becoming more widely accepted because it is effective in treating a variety of shoulder conditions. In MWM, a patient applies a continuous glide force while simultaneously performing an active movement (active movement component) [15].

According to Teys, kinematic assessments of patients with excessive superior and/or anterior translation of the humeral head in the glenoid fossa have been shown. These patients have impingement, RCTs, loss of capsuloligamentous integrity, and neuromuscular fatigue. It has been proposed that this error can be fixed, and ideal pain-free motion can be achieved by applying a posterior glide MWM to the shoulder [16]. In a study conducted by Stathopoulos, an effective therapeutic outcome requires many repetitions of the pain-free MWM, together with the extra application of overpressure. Peripheral joint mobilization directions include anteroposterior and posteroanterior glide, internal and external rotation, and internal and external lateral glide [17].

## Conclusions

For patients with RC tendinopathy, an optimal rehabilitation routine that encompasses favorable long-term recovery was given. This case report describes a farmer who suffered from RC tendinopathy and underwent physiotherapy rehabilitation. He had physiotherapy treatments that fulfilled his expectations and were satisfactory. The patient reported that his upper limb functional activity in ROM, strength of the muscles surrounding the shoulder, activities of daily living (ADL), and functional independence have improved. We believe that this case report contributes to our expertise concerning how these patients should be treated.
